# *pOsNAR2.1:OsNAR2.1* expression enhances nitrogen uptake efficiency and grain yield in transgenic rice plants

**DOI:** 10.1111/pbi.12714

**Published:** 2017-03-29

**Authors:** Jingguang Chen, Xiaoru Fan, Kaiyun Qian, Yong Zhang, Miaoquan Song, Yu Liu, Guohua Xu, Xiaorong Fan

**Affiliations:** ^1^ State Key Laboratory of Crop Genetics and Germplasm Enhancement Nanjing Agricultural University Nanjing China; ^2^ Key Laboratory of Plant Nutrition and Fertilization in Low‐Middle Reaches of the Yangtze River Ministry of Agriculture Nanjing Agricultural University Nanjing China; ^3^ State Key Laboratory of Plant Physiology and Biochemistry College of Life Science Zhejiang University Hangzhou China

**Keywords:** *OsNAR2.1* promoter, *OsNAR2.1*, *Oryza sativa*, Nitrogen uptake efficiency

## Abstract

The nitrate (${\text{NO}}_{3}^{-}$) transporter has been selected as an important gene maker in the process of environmental adoption in rice cultivars. In this work, we transferred another native *OsNAR2.1* promoter with driving *OsNAR2.1* gene into rice plants. The transgenic lines with exogenous *pOsNAR2.1:OsNAR2.1* constructs showed enhanced *OsNAR2.1* expression level, compared with wild type (WT), and ^15^N influx in roots increased 21%–32% in response to 0.2 mm and 2.5 mm 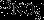 and 1.25 mm ^15^ NH_4_^15^ NO_3_. Under these three N conditions, the biomass of the *pOsNAR2.1:OsNAR2.1* transgenic lines increased 143%, 129% and 51%, and total N content increased 161%, 242% and 69%, respectively, compared to WT. Furthermore in field experiments we found the grain yield, agricultural nitrogen use efficiency (ANUE), and dry matter transfer of *pOsNAR2.1:OsNAR2.1* plants increased by about 21%, 22% and 21%, compared to WT. We also compared the phenotypes of *pOsNAR2.1:OsNAR2.1* and *pOsNAR2.1:OsNRT2.1* transgenic lines in the field, found that postanthesis N uptake differed significantly between them, and in comparison with the WT. Postanthesis N uptake (PANU) increased approximately 39% and 85%, in the *pOsNAR2.1:OsNAR2.1* and *pOsNAR2.1:OsNRT2.1* transgenic lines, respectively, possibly because *OsNRT2.1* expression was less in the *pOsNAR2.1:OsNAR2.1* lines than in the *pOsNAR2.1:OsNRT2.1* lines during the late growth stage. These results show that rice NO_3_^–^ uptake, yield and NUE were improved by increased *OsNAR2.1* expression *via* its native promoter.

## Introduction

Nitrogen (N) is an essential macronutrient for plant growth and crop productivity. ${\text{NO}}_{3}^{-}$ is the main inorganic N nutrient for plants in aerobic uplands, and ${\text{NH}}_{4}^{+}$ is the main form in anaerobic paddy fields (Foyer *et al*., 1998; Scheible *et al*., 2004; Stitt, 1999). In upland cultivation system, ${\text{NO}}_{3}^{-}$ is readily dissolved in soil water and very mobile in soil and therefore it was very easily lost into environment (Jin *et al*., 2015; Zarabi and Jalali, 2012). ${\text{NO}}_{3}^{-}$ is acquired by roots through ${\text{NO}}_{3}^{-}$ transporters and then transported throughout the plant, or it can be assimilated with carbon into amino acids before being redistributed (Katayama *et al*., 2009; Miller *et al*., 2007; Xu *et al*., 2012). In plants, seed dormancy can be broken by ${\text{NO}}_{3}^{-}$ as a signalling molecule (Alboresi *et al*., 2005; Matakiadis *et al*., 2009), regulating lateral root development (Zhang and Forde, 1998; Zhang *et al*., 1999) and leaf growth (Hsu and Tsay, 2013; Rahayu *et al*., 2005), integrating the expression of nitrate‐induced genes for growth and development (Dechorgnat *et al*., 2012; Ho and Tsay, 2010; Huang *et al*., 2015; O'Brien *et al*., 2016; Wang *et al*., 2012) and altering flowering time (Castro Marin *et al*., 2011).

As for adapting to the low and high ${\text{NO}}_{3}^{-}$ concentrations in soil, the plants have developed two different absorption systems (Léran *et al*., 2014; Miller *et al*., 2007; Siddiqi *et al*., 1990), including the low ${\text{NO}}_{3}^{-}$ affinity system (LATS) and high ${\text{NO}}_{3}^{-}$ affinity system (HATS) (Crawford and Glass, 1998). As we know the NPF (NRT1/PTR) and NRT2 families contribute to LATS and HATS responding the ${\text{NO}}_{3}^{-}$ uptake and translocation in plants (Fan *et al*., 2005; Léran *et al*., 2014; Miller *et al*., 2007; Orsel *et al*., 2006; Szczerba *et al*., 2006).

Some NRT2 family members in plant are needed NAR2 partners in transporting nitrate crossing cell membrane (Galván *et al*., 1996; Liu *et al*., 2014; Okamoto *et al*., 2006; Orsel *et al*., 2006; Quesada *et al*., 1994; Tong *et al*., 2005; Zhuo *et al*., 1999). In *Chlamydomonas reinhardtii* Quesada *et al*. (1994) firstly found that CrNar2 and CrNar3 can restore ${\text{NO}}_{3}^{-}$ absorption of the ${\text{NO}}_{3}^{-}$ uptake‐defective mutants. Zhou *et al*. (2000) further demonstrated that CrNar2 was a partner protein of CrNRT2.1 in ${\text{NO}}_{3}^{-}$ transporting cross the oocyte cell membrane. Okamoto *et al*. (2006) reported that, based on NAR2‐type gene expression, both NAR2s and NRT2s constitute the ${\text{NO}}_{3}^{-}$ inducible HATS, but not the LATS in Arabidopsis, such as AtNRT3, although the protein had no known transport activity. Yong *et al*. (2010) reported that *in vivo* NAR2.1 and NRT2.1 forming a complex on plasma membrane and played the role in absorbing low concentration of nitrate in Arabidopsis roots. Orsel *et al*. (2006) used oocyte expression and yeast split‐ubiquitin systems to show that AtNAR2.1 and AtNRT2.1 are partners in a two‐component HATS.

Two‐component NRT2‐NAR2 system also exists in rice ${\text{NO}}_{3}^{-}$ transport process. Feng *et al*. (2011) used an oocyte expression system to show that only OsNAR2.1, but not OsNAR2.2, interacts with OsNRT2.3a or OsNRT2.1/2.2 to promote ${\text{NO}}_{3}^{-}$ uptake. Katayama *et al*. (2009) reported that overexpression of *OsNRT2.1* improved the growth of rice seedlings, but did not increase nitrogen uptake. Tang *et al*. (2012) showed that rice *OsNRT2.3a* gene is involved in root transport of ${\text{NO}}_{3}^{-}$ to shoots. The OsNRT2.3a or OsNRT2.1/2.2 and OsNAR2.1 interaction at the protein level was demonstrated using bimolecular fluorescence complementation, the yeast two‐hybrid system and Western blot analysis (Liu *et al*., 2014; Yan *et al*., 2011). Furthermore Yan *et al*. (2011) also reported that knockdown of *OsNAR2.1* by RNA interference (RNAi) can suppress expression of *OsNRT2.3a*,* OsNRT2.2* and *OsNRT2.1* in mutants roots and demonstrated that *OsNAR2.1* does a key function in both high and low ${\text{NO}}_{3}^{-}$ uptake.

Chen *et al*. (2016) showed that using *OsNAR2.1* promoter instead of ubiquitin promoter driving *OsNRT2.1* can improve the ANUE and yield in rice. In this study, we created new construct of *OsNAR2.1* promoter to drive the open reading frame (ORF) of the *OsNAR2.1,* investigated the transformation effects of *pOsNAR2.1:OsNAR2.1* on rice ${\text{NO}}_{3}^{-}$ uptake, yield and NUE and also presented many different characteristics of *pOsNAR2.1:OsNAR2.1* from *pOsNAR2.1:OsNRT2.1* transgenic plants.

## Results

### Generation of transgenic rice expressing *pOsNAR2.1:OsNAR2.1*

**Figure 1:**
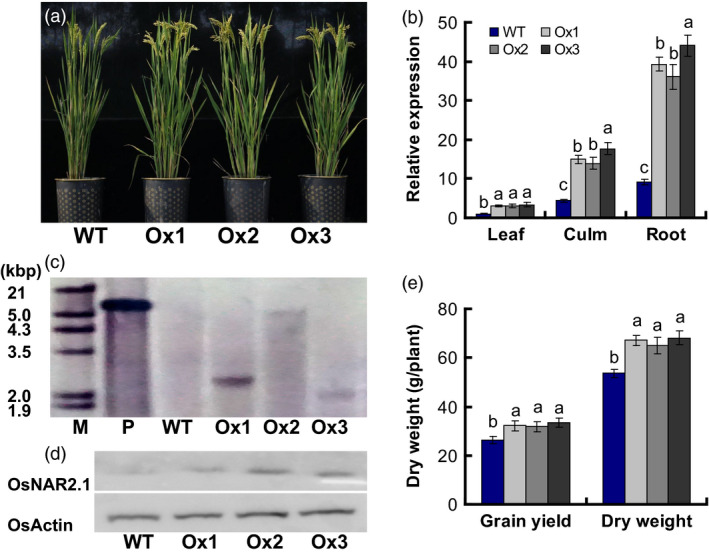
Characterization of *pOsNAR2.1:OsNAR2.1* transgenic lines. (a) Phenotype of wild‐type and *pOsNAR2.1:OsNAR2.1* transgenic plants (Ox1, Ox2 and Ox3). (b) qRT‐PCR analysis the expression of *OsNAR2.1*. RNA was extracted from root, culm and Leaf blade I. Error bars: SE (*n* = 3 plants). (c) Southern blot of genomic DNA isolated from T2 generation transgenic plants and WT. Hybridization using a hygromycin gene probe. P, positive control, M, marker. (d) Western blot of total proteins from shoots of T2 generation transgenic plants and WT. Hybridization with an OsActin‐specific antibody and an OsNAR2.1‐specific antibody. Each lane was loaded with equal quantity of protein (50 μg). (e) Biomass and grain yield per plant in the field. Error bars: SE (*n* = 5 plants). The different letters indicate a significant difference between the transgenic line and the WT (*P *< 0.05, one‐way ANOVA).

We used the *Agrobacterium tumefaciens*‐mediated method to introduce the *pOsNAR2.1:OsNAR2.1* expression construct (Figure S1) into Wuyunjing 7 (*O. sativa* L. ssp. Japonica cv., the wild type for this experiment, WT), a high yield rice cultivar used in Jiangsu, China. We obtained 10 lines with increased the expression of *OsNAR2.1* (Figure S2a) and analysed biomass and yield of the transgenic plants in the T1 generation. Compared to WT, biomass and yield of the 10 lines of T1 generation increased by approximately 13% and 20%, respectively (Figure S2b). Based on a Southern blot analysis of T2 generation and the data of RNA expression for the T1 and T2 generations (Figures 1c, S2a and 1b), we selected three independent lines of *pOsNAR2.1:OsNAR2.1* designated Ox1, Ox2 and Ox3 (Figure 1a).

The expression of *OsNAR2.1* in roots was increased four‐ to fivefold in the Ox1, Ox2 and Ox3 lines. *OsNAR2.1* expression increased approximately 3.5‐fold in culms and increased approximately 2.6‐fold in leaf blades of the *pOsNAR2.1:OsNAR2.1* transgenic plants (Figure 1b). The Western blot showed that the protein level of OsNAR2.1 was increased in shoots of Ox1, Ox2 and Ox3 lines compared with WT (Figure 1d). The field data showed that the transgenic lines exhibited increased grain yield and dry weight, compared with the WT (Figures 1e and S2b). Field data of the T2, T3 and T4 generation lines showed that total aboveground biomass, increased by as much as 23%; yields of T3 transgenic plants grown at Sanya were enhanced by approximately 20%, and the yields of T2 and T4 plants grown at Nanjing increased by 21%–23%, relative to the WT (Table S3).

For the T4 transgenic plants at harvest, height increased 5%, total tiller number per plant increased 26%, panicle length increased approximately 12%, grain weight per panicle increased 25%, seed setting rate increased 13%, grain number per panicle increased 16%, and grain yields increased by 23% relative to the WT; however, 1000‐grain weight had no difference between WT and the transgenic lines (Table 1).

**Table 1:** Comparison of agronomic traits of *pOsNAR2.1:OsNAR2.1* transgenic lines

Genotype	WT	Ox1	Ox2	Ox3
Plant height (cm)	83.81b	87.74a	87.22a	88.15a
Total tiller number per plant	20.48b	26.78a	25.14a	25.46a
Panicle length (cm)	13.78b	15.67a	15.24a	15.56a
Grain number per panicle	130.67b	153.80a	149.56a	152.66a
Seed setting rate (%)	72.67b	83.04a	80.33a	82.45a
Grain weight (g/panicle)	2.32b	3.01a	2.77a	2.89a
1000‐grain weight (g)	25.79a	25.65a	25.87a	25.74a
Grain yield (g/plant)	26.37b	32.14a	31.81a	33.38a

Statistical analysis of data from T4 generation; *n* = 3 plots for each mean. The different letters indicate a significant difference between the transgenic line and the WT. (*P* < 0.05, one‐way ANOVA).

### Effects of *pOsNAR2.1:OsNAR2.1* expression on plant seedling growth and total nitrogen content

**Figure 2:**
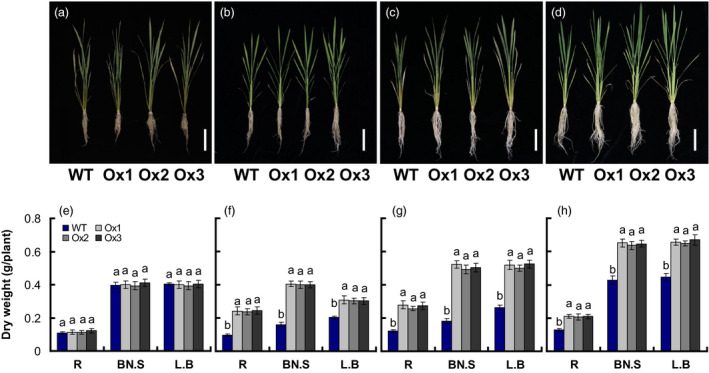
Comparison of growth of *pOsNAR2.1:OsNAR2.1* transgenic lines at different nitrogen supply levels. WT and transgenic rice seedlings in the solution containing 1 mm ${\text{NH}}_{4}^{+}$ of IRRI for 2 weeks and then in different forms of nitrogen for 3 additional weeks. Phenotype of the *pOsNAR2.1:OsNAR2.1* lines (Ox1, Ox2 and Ox3) grown with (a) 2.5 mm ${\text{NH}}_{4}^{+}$, (b) 0.2 mm ${\text{NO}}_{3}^{-}$, (c) 2.5 mm ${\text{NO}}_{3}^{-}$ and (d) 1.25 mm NH_4_ NO_3_; bar = 10 mm; dry weight of seedlings treated with (e) 2.5 mm ${\text{NH}}_{4}^{+}$, (f) 0.2 mm ${\text{NO}}_{3}^{-}$, (g) 2.5 mm ${\text{NO}}_{3}^{-}$ and (h) 1.25 mm NH_4_ NO_3_. L.B, leaf blade; BN.S, basal node and sheath; R, root. Error bars: SE (*n* = 4 plants). The different letters indicate a significant difference between the transgenic line and the WT (*P* < 0.05, one‐way ANOVA).

As previous data showed that knockdown of *OsNAR2.1* in rice affects N uptake and growth (Yan *et al*., 2011). We further analysed the effect of *pOsNAR2.1:OsNAR2.1* expression on plant seedling growth and nitrogen content by planting WT and transgenic rice seedlings in the solution containing 1 mm ${\text{NH}}_{4}^{+}$ of IRRI for 2 weeks and then in 2.5 mm ${\text{NH}}_{4}^{+}$, 0.2 mm ${\text{NO}}_{3}^{-}$, 2.5 mm ${\text{NO}}_{3}^{-}$ or 1.25 mm NH_4_NO_3_ for 3 more weeks (Figure 2a–d). While the dry weight of roots, leaf sheaths and leaves of the *pOsNAR2.1:OsNAR2.1* transgenic line were not affected by growth in 2.5 mm ${\text{NH}}_{4}^{+}$ (Figure 2e), they increased, respectively, by 152%, 149% and 151% in 0.2 mm ${\text{NO}}_{3}^{-}$ (Figure 2f); by 124%, 181% and 95% in 2.5 mm ${\text{NO}}_{3}^{-}$ (Figure 2g); and by 62%, 51% and 47% in 1.25 mm NH_4_NO_3_, compared with WT after harvest (Figure 2h).

**Figure 3:**
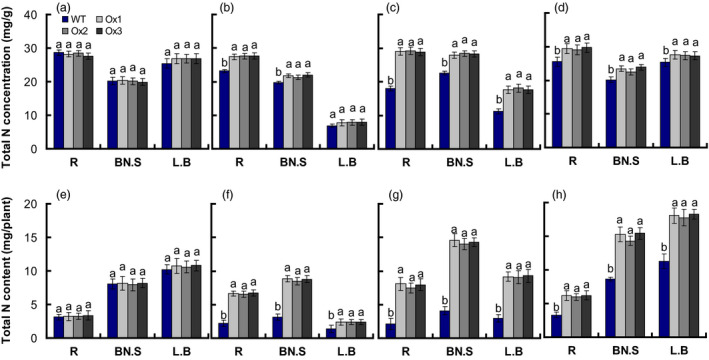
Comparison of total nitrogen concentration and total nitrogen content of *pOsNAR2.1:OsNAR2.1* transgenic plants at different nitrogen supply levels. WT and transgenic rice seedlings in the solution containing 1 mm ${\text{NH}}_{4}^{+}$ of IRRI for 2 weeks, and in different forms of nitrogen for 3 additional weeks. Total nitrogen concentration of seedlings treated with (a) 2.5 mm ${\text{NH}}_{4}^{+}$, (b) 0.2 mm ${\text{NO}}_{3}^{-}$, (c) 2.5 mm ${\text{NO}}_{3}^{-}$ and (d) 1.25 mm NH_4_ NO_3_; Total N content of seedlings grown with (e) 2.5 mm ${\text{NH}}_{4}^{+}$, (f) 0.2 mm ${\text{NO}}_{3}^{-}$, (g) 2.5 mm ${\text{NO}}_{3}^{-}$ and (h) 1.25 mm NH_4_ NO_3_. L.B, leaf blade; BN.S, basal node and sheath; R, root. Error bars: SE (*n* = 4 plants). The different letters indicate a significant difference between the transgenic line and the WT (*P* < 0.05, one‐way ANOVA).

Total N concentrations of roots, leaf sheaths and leaves in *pOsNAR2.1:OsNAR2.1* were not affected by 2.5 mm ${\text{NH}}_{4}^{+}$ (Figure 3a), but were increased by 19%, 10% and 14%, in 0.2 mm ${\text{NO}}_{3}^{-}$ (Figure 3b); by 62%, 25% and 60% in 2.5 mm ${\text{NO}}_{3}^{-}$ (Figure 3c); and by 15%, 15% and 8% in 1.25 mm NH_4_NO_3_ (Figure 3d), respectively. Total N contents of roots, leaf sheaths and leaves in *pOsNAR2.1:OsNAR2.1* were not affected by 2.5 mm ${\text{NH}}_{4}^{+}$ (Figure 3e), but were increased by 199%, 174% and 72%, in 0.2 mm ${\text{NO}}_{3}^{-}$ (Figure 3f); by 263%, 251% and 212% in 2.5 mm ${\text{NO}}_{3}^{-}$ (Figure 3g); and by 87%, 74 and 60% in 1.25 mm NH_4_NO_3,_ compared with WT (Figure 3h), respectively.

**Figure 4:**
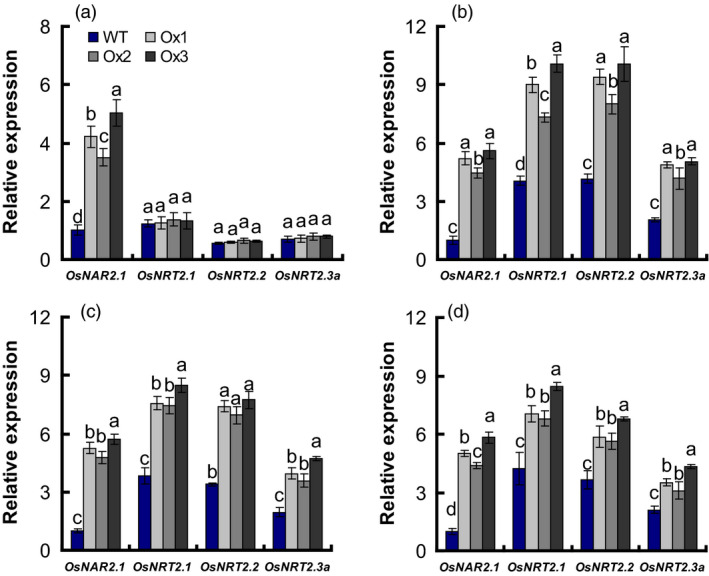
The expression of *OsNRT2s* and *OsNAR2.1* in *pOsNAR2.1:OsNAR2.1* transgenic lines. Extraction of total RNA from roots of WT and transgenic lines as showing in Figure 2 and qRT‐PCR result under (a) 2.5 mm ${\text{NH}}_{4}^{+}$, (b) 0.2 mm ${\text{NO}}_{3}^{-}$, (c) 2.5 mm ${\text{NO}}_{3}^{-}$ and (d) 1.25 mm NH_4_ NO_3_ conditions. Error bars: SE (*n* = 3 plants). The different letters indicate a significant difference between the transgenic line and the WT (*P* < 0.05, one‐way ANOVA).

Yan *et al*. (2011) reported that *OsNAR2.1* RNAi affects the expression of interacting proteins with the *OsNAR2.1* including *OsNRT2.1*,* OsNRT2.2* and *OsNRT2.3a* genes. We further analysed whether *OsNAR2.1* and *OsNRT2s* expression in transgenic rice roots was altered at differing N supply rates. Transcription of *OsNRT2.3a*,* OsNRT2.2* and *OsNRT2.1* in transgenic plant roots was not affected by growth in 2.5 mm ${\text{NH}}_{4}^{+}$ (Figure 4a); but was increased, respectively, by 117, 121 and 129% in 0.2 mm ${\text{NO}}_{3}^{-}$ (Figure 4b); by 105%, 118% and 110%, in 2.5 mm ${\text{NO}}_{3}^{-}$ (Figure 4c); and by 76%, 68% and 73% in 1.25 mm NH_4_NO_3_ (Figure 4d), compared with WT.

### Rates of ${\text{NO}}_{3}^{-}$ and ${\text{NH}}_{4}^{+}$ influx in WT and transgenic plants

**Figure 5:**
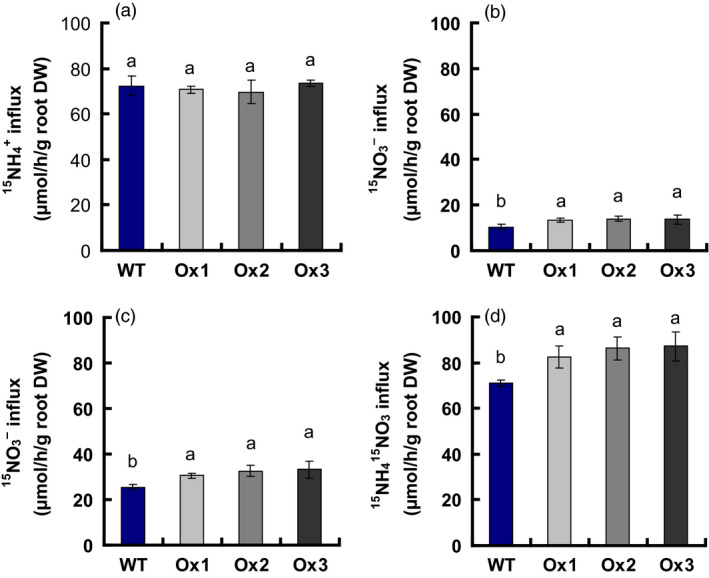
${\text{NH}}_{4}^{+}$ and ${\text{NO}}_{3}^{-}$ influx rates of *pOsNAR2.1:OsNAR2.1* transgenic lines measured using ^15^N‐enriched sources. WT and transgenic seedlings were grown in 1 mm ${\text{NH}}_{4}^{+}$ for 3 weeks and nitrogen starved for 1 week. ^15^N influx rates were then measured at (a) 2.5 mm 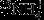, (b) 0.2 mm ^15^ NO_3_^–^, (c) 2.5 mm ^15^ NO_3_^–^ and (d) 1.25 mm ^15^ NH_4_^15^ NO_3_ during 5 min. DW, dry weight. Error bars: SE (*n* = 4 plants). The different letters indicate a significant difference between the transgenic line and the WT (*P* < 0.05, one‐way ANOVA).

We analysed short‐term ${\text{NO}}_{3}^{-}$ and ${\text{NH}}_{4}^{+}$ uptake in same‐size seedlings of the *pOsNAR2.1:OsNAR2.1* transgenic lines and WT by exposing the plants to 2.5 mm 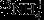, 0.2 mm 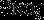, 2.5 mm 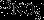, 1.25 mm ^15^NH_4_^15^NO_3_, 1.25 mm ^15^NH_4_NO_3_ or 1.25 mm NH_4_^15^NO_3_ for 5 min to determine the effect of *pOsNAR2.1:OsNAR2.1* expression on root ${\text{NO}}_{3}^{-}$ and ${\text{NH}}_{4}^{+}$ influx into intact plants. The influx rate of 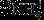 in the Ox1, Ox2 and Ox3 transgenic lines did not change compared with that of WT (Figure 5a); however, the influx rate of 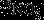 increased 32% and 26% in response to 0.2 mm 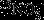 and 2.5 mm 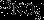, respectively, in the *pOsNAR2.1:OsNAR2.1* transgenic lines (Figure 5b, c). The influx rate of ^15^NH_4_^15^NO_3_ in the transgenic lines increased about 20% in 1.25 mm ^15^NH_4_^15^NO_3_ (Figure 5d), and the influx rates of 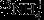 and 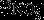 increased by 21% and 22% in 1.25 mm ^15^NH_4_NO_3_ and 1.25 mm NH_4_^15^NO_3_, respectively (Figure S3a, b). The ratio of 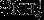 to 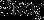 influx in *pOsNAR2.1:OsNAR2.1* transgenic and WT plants did not differ in response to 1.25 mm ^15^NH_4_NO_3_ and 1.25 mm NH_4_^15^NO_3_ (Figure S3c).

### Translocation of dry matter and nitrogen in WT and transgenic plants

**Figure 6:**
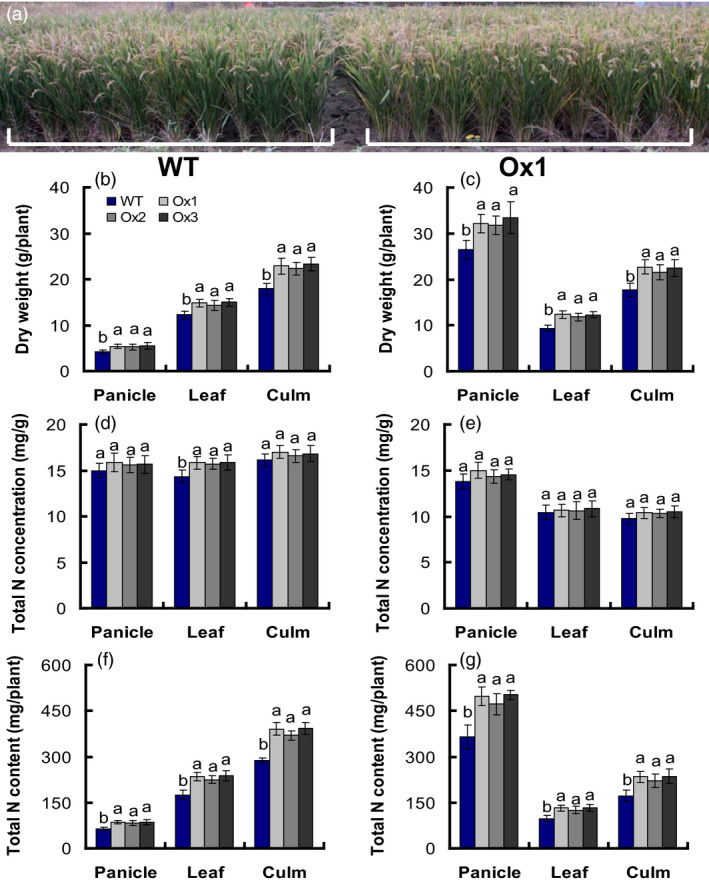
Biomass and nitrogen content in different parts of *pOsNAR2.1:OsNAR2.1* transgenic lines at the anthesis stage and maturity stage. (a) Photograph of WT and T4 generation Ox1 in the field experiment. Biomass in various parts of WT and T4 generation transgenic plants at (b) the anthesis stage and (c) maturity stage. Nitrogen concentration in different parts of transgenic lines and WT at the (d) anthesis stage and (e) maturity stage. Nitrogen content in different parts of transgenic lines and WT at the (f) anthesis stage and (g) maturity stage. Error bars: SE (*n* = 5 plants). The different letters indicate a significant difference between the transgenic line and the WT (*P* < 0.05, one‐way ANOVA).

Methods to measure NUE usually depend on calculating plant biomass production per unit of applied N, regardless of the crop and whether the root, leaf, fruit, or seed is measured, the transfer of N to plant organs and yield is known as “nutrient utilization efficiency” (Good *et al*., 2004; Xu *et al*., 2012). We analysed the dry matter, total nitrogen concentration and the total nitrogen content of the T4 generation of the *pOsNAR2.1:OsNAR2.1* transgenic lines in the anthesis and maturity stages. The result showed that the biomass of panicles, leaves and culms in the transgenic lines increased 26%, 20% and 28%, respectively, in the anthesis stage (Figure 6b), and increased 23%, 29% and 25% in the maturity stage compared to those of WT (Figure 6c). Total nitrogen concentration in leaves of the transgenic lines increased approximately 10% in the anthesis stage, but did not change in panicles or culms compared to those of WT. Total nitrogen concentration of panicles, leaves and culms was not different at the maturity stage compared to that in WT (Figure 6e); total nitrogen content of panicles, leaves and culms in the transgenic lines increased by approximately 34%, 33% and 33%, respectively, during the anthesis stage (Figure 6f), and by 35%, 33% and 34% in the maturity stage, respectively, compared to those in the WT (Figure 6f).

We calculated nitrogen and dry matter translocation in plants by determining dry matter at maturity (DMM), dry matter at anthesis (DMA), grain yield (GY), total nitrogen accumulation at maturity (TNAM), grain nitrogen accumulation at maturity (GNAM) and total nitrogen accumulation at anthesis (TNAA). Compared to the WT, DMA, DMM, GY, TNAA, TNAM and GNAM increased by approximately 25%, 25%, 24%, 33%, 34% and 35%, respectively, in *pOsNAR2.1:OsNAR2.1* transgenic plants (Table 2).

**Table 2:** Biomass and nitrogen content of *pOsNAR2.1:OsNAR2.1* transgenic lines

Dry matter and nitrogen components:	WT	Ox1	Ox2	Ox3
DMA (kg/m^2^)	0.86b	1.08a	1.05a	1.09a
DMM (kg/m^2^)	1.34b	1.68a	1.63a	1.70a
GY (kg/m^2^)	0.66b	0.81a	0.80a	0.84a
TNAA (g/m^2^)	13.17b	17.76a	16.90a	17.90a
TNAM (g/m^2^)	15.87b	21.61a	20.46a	21.74a
GNAM (g/m^2^)	9.11b	12.44a	11.79a	12.55a

Statistical analysis of data from T4 generation; *n* = 3 plots for each mean. The different letters indicate a significant difference between the transgenic line and the WT (*P* < 0.05, one‐way ANOVA).

We also calculated the harvest index (HI), dry matter translocation (DMT), dry matter translocation efficiency (DMTE) and the contribution of pre‐anthesis assimilates to grain yield (CPAY), based on a method described by Chen *et al*. (2016). DMT increased by approximately 21%, whereas DMTE, CPAY and HI had no difference in *pOsNAR2.1:OsNAR2.1* transgenic plants from WT (Table 3). We investigated nitrogen translocation (NT), contribution of pre‐anthesis nitrogen to grain nitrogen accumulation (CPNGN) and NT efficiency (NTE), based on a method described by Chen *et al*. (2016). NTE and CPNGN did not differ *pOsNAR2.1:OsNAR2.1* transgenic plants from WT, whereas NT increased by approximately 33%, relative to that in WT (Table 3).

**Table 3:** DMT and NT of the *pOsNAR2.1:OsNAR2.1* transgenic lines

	WT	Ox1	Ox2	Ox3
PNUE (g/g)	51.77a	50.16a	51.71a	52.15a
NHI (%)	57.36a	57.58a	57.62a	57.70a
DMT (g/m^2^)	182.32b	223.39a	213.80a	226.97a
DMTE (%)	21.20a	18.86a	20.43a	20.74a
CPAGY (%)	27.62a	27.34a	26.89a	27.18a
HI (%)	49.35a	48.84a	48.85a	49.05a
NT (g/m^2^)	6.40b	8.59a	8.23a	8.69a
NTE (%)	48.61a	48.39a	48.70a	48.57a
CPNGN (%)	70.32a	69.38a	69.82a	69.24a

PNUE (kg/kg) = (GY – GY of zero‐N plot)/TNAM; NHI (%) = (GNAM/TNAM) × 100%; DMT (kg/ha) = DMA – (DMM – GY); DMTE (%) = (DMT/DMA) × 100%; CPAY (%) = (DMT/GY) × 100%; HI (%) = (GY/DMM) × 100%; NT (kg/ha) = TNAA – (TNAM – GNAM); NTE (%) = (NT/TNAA) × 100%; CPNGN (%) = (NT/GNAM) × 100%. Statistical analysis of data from T4 generation; *n* = 3 plots for each mean. The different letters indicate a significant difference between the transgenic line and the WT (*P* < 0.05, one‐way ANOVA).

### NUE of *pOsNAR2.1:OsNAR2.1* transgenic lines

**Figure 7:**
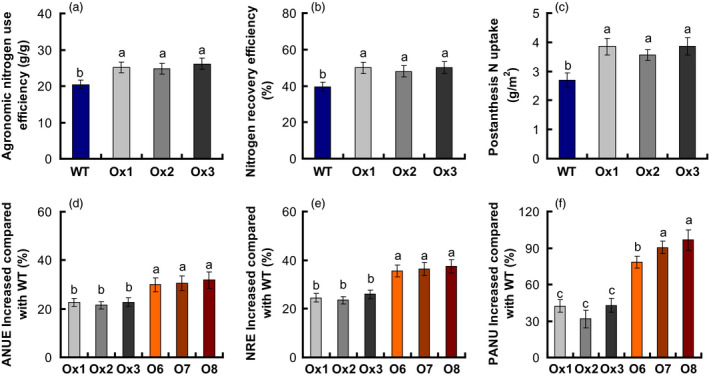
Increased nitrogen use efficiency (NUE) in *pOsNAR2.1:OsNAR2.1* and *pOsNAR2.1:OsNRT2.1* transgenic plants relative to wild type. Comparison of (a) agronomic nitrogen use efficiency (ANUE), (b) nitrogen recovery efficiency (NRE) and (c) postanthesis N uptake (PANU) between *pOsNAR2.1:OsNAR2.1* transgenic lines and wild type. Enhanced percentage of (d) ANUE, (e) NRE and (f) PANU of *pOsNAR2.1:OsNAR2.1* and *pOsNAR2.1:OsNRT2.1* relative to wild type. *n* = 3 plots for each mean. The different letters indicate a significant difference between the transgenic line and the WT (*P* < 0.05, one‐way ANOVA).

NUE is inherently compound and can be further defined with component parts, including NUpE, NUtE, ANR, AE NTE, NRE (Xu *et al*., 2012). Because both yield and biomass were increased in the *pOsNAR2.1:OsNAR2.1* transgenic lines, in the meanwhile, we also investigated ANUE in transgenic plants of T2–T4 generations, and nitrogen recovery efficiency (NRE), PANU, nitrogen harvest index (NHI), and physiological nitrogen use efficiency (PNUE) traits in the T4 transgenic plants to determine whether nitrogen use was changed in these lines, using the method described by Chen *et al*. (2016). Compared to WT, the ANUE of the *pOsNAR2.1:OsNAR2.1* transgenic lines was enhanced by approximately 22% in T3 generation grown at Sanya under the tropical climate condition and by 21%–24% in the T2 and T4 plants grown at Nanjing under semi‐tropical condition (Table S3, Figure 7a). NRE and PANU increased approximately 125% and 39% in the T4 generations, compared to those in WT (Figure 7b, c), but PNUE and NHI values had no different between those and WT (Table 3).

### Comparison between NUEs in *pOsNAR2.1:OsNAR2.1* and *pOsNAR2.1:OsNRT2.1* transgenic plants

In the field, PNUE, NHI, DMTE, CPAY, HI and NTE values of *pOsNAR2.1:OsNAR2.1* and *pOsNAR2.1:OsNRT2.1* transgenic lines were the same as those of WT plants (Table S4). Compared to WT, ANUE increased approximately 22% and 31%, in the *pOsNAR2.1:OsNAR2.1* and *pOsNAR2.1:OsNRT2.1* transgenic lines, respectively. NRE increased approximately 25% and 36%, and PANU increased approximately 39 and 85% (Figure 7d–f). The CPNGN of *pOsNAR2.1:OsNAR2.1* transgenic lines showed no difference compared with WT, but the CPNGN decreased about 15% in *pOsNAR2.1:OsNRT2.1* lines (Table S4).

### The expression of *OsNRT2.1* and *OsNAR2.1* in transgenic lines

**Figure 8:**
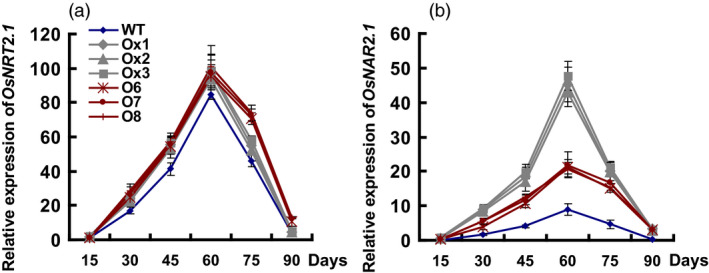
Expression of *OsNRT2.1* and *OsNAR2.1* in transgenic lines and wild type during the experimental growth period. RNA Samples of T4 generation plant culms were collected every 15 days, from the beginning of rice transplant to mature stage. Error bars: SE (*n* = 3 plants). D in *x*‐axis means the day after transplanting.

The expression levels of *OsNRT2.1* and *OsNAR2.1* in culms were significantly increased in all transgenic lines, compared to the WT plants (Figure 8a, b). The expression of *OsNRT2.1* was about 32% and 38% higher in the *pOsNAR2.1:OsNAR2.1* and *pOsNAR2.1:OsNRT2.1* lines than in WT (Figure 8a). The expression of *OsNAR2.1* was 4.1–6.4‐fold higher in the *pOsNAR2.1:OsNAR2.1* lines than in WT and 2.3–3.6‐fold higher in the *pOsNAR2.1:OsNRT2.1* lines than in WT (Figure 8b). And the expression of *OsNRT2.1* showed no difference between the *pOsNAR2.1:OsNAR2.1* and *pOsNAR2.1:OsNRT2.1* lines throughout the experimental growth period, except at 75 days (Figure 8a). The expression of *OsNAR2.1* was significantly higher in the culms of the *pOsNAR2.1:OsNAR2.1* lines than of the *pOsNAR2.1:OsNRT2.1* lines (Figure 8b).

We also calculated the expression ratio of *OsNRT2.1* and *OsNAR2.1* in different plants, which was approximately 2.2:1 in the *pOsNAR2.1:OsNAR2.1* lines, 4.6:1 in the *pOsNAR2.1:OsNRT2.1* lines and 10.6:1 in WT (Figure S4).

## Discussion

All levels of plant function were affected by nitrogen nutrition, from metabolism to growth, development and resource allocation (Crawford, 1995; Scheible *et al*., 1997). ${\text{NO}}_{3}^{-}$ is a main available form of nitrogen for plants and is absorbed in the roots by active transport processes and passive transport ion channels, stored in vacuoles of rice shoots (Fan *et al*., 2007; Kucera, 2003; Li *et al*., 2008; Pouliquin *et al*., 2000). OsNRT2.1/2 and OsNRT2.3a need to be combined with OsNAR2.1 protein for uptake and transport of ${\text{NO}}_{3}^{-}$ in rice (Liu *et al*., 2014; Tang *et al*., 2012; Yan *et al*., 2011). The expression of *OsNAR2.1* is up‐regulated by ${\text{NO}}_{3}^{-}$ and down‐regulated by ${\text{NH}}_{4}^{+}$ (Feng *et al*., 2011; Yan *et al*., 2011).

Feng *et al*. (2011) reported that the native *OsNAR2.1* promoter has strong activities in roots and basal nodes in seedlings. In this study, *OsNAR2.1* expression was up‐regulated significantly in both roots and shoots of *pOsNAR2.1:OsNAR2.1* transgenic lines (Figure 1b). Previous report had addressed the *OsNAR2.1* promoter induction by ${\text{NO}}_{3}^{-}$ in rice based on GUS fusion data (Feng *et al*., 2011); Yan *et al*. (2011) reported the effect of rice seedling stage and nitrogen uptake on growth after OsNAR2.1 knockdown; moreover Chen *et al*. (2016) reported the gain function of *pOsNAR2.1:OsNRT2.1* expression on rice growth and nitrogen use. Here, we focused on nitrogen uptake and growth at the seedling stage, field yield and NUE in *pOsNAR2.1:OsNAR2.1* transgenic lines.

### *pOsNAR2.1:OsNAR2.1* expression increases ${\text{NO}}_{3}^{-}$ uptake of transgenic rice plants

Feng *et al*. (2011) had proved that *OsNAR2.1* interacts with *OsNRT2.3a* and *OsNRT2.1/2.2* in an oocyte expression system to take up ${\text{NO}}_{3}^{-}$. The OsNAR2.1 and OsNRT2.3a or OsNRT2.1/2.2 interaction in the protein level was demonstrated using bimolecular fluorescence complementation, Western blot analysis and a yeast two‐hybrid assay (Liu *et al*., 2014; Yan *et al*., 2011). Tang *et al*. (2012) showed that *OsNRT2.3a* gene is important in ${\text{NO}}_{3}^{-}$ root transport to shoots. Katayama *et al*. (2009) reported that increased *OsNRT2.1* expression slightly improved the growth of rice seedling in hydroponic condition, but did not affect the nitrogen uptake. In this study, we demonstrated that *OsNAR2.1* driven by the native *OsNAR2.1* promoter increased ${\text{NO}}_{3}^{-}$ uptake by rice roots.

As we know, the native *OsNAR2.1* was expressed in all parts in rice plant, and mainly expressed roots and leaf sheaths (Chen *et al*., 2016; Feng *et al*., 2011; Liu *et al*., 2014; Yan *et al*., 2011), but we do not know why one more native promoter driving *OsNAR2.1* can increase the expression level of *OsNAR2.1* more than one time and in different organs, the increase patterns were different. The possible reason about this was that the methylation level was different in the transferred homologous exogenous promoter sequence compared with the endogenous promoter sequence (Matzke *et al*., 1989). However more experiments are needed for this understanding.

Rice dry weight and total nitrogen content of *pOsNAR2.1:OsNAR2.1* transgenic plants differed clearly from WT plants when the plants were supplied with 1.25 mm NH_4_NO_3_, 0.2 mm ${\text{NO}}_{3}^{-}$ or 2.5 mm ${\text{NO}}_{3}^{-}$ (Figures 2 and 3). *OsNRT2.3a*,* OsNRT2.2 and OsNRT2.1* expression which encode OsNAR2.1*‐*interacting proteins, increased significantly in 1.25 mm NH_4_NO_3_, 0.2 mm ${\text{NO}}_{3}^{-}$ and 2.5 mm ${\text{NO}}_{3}^{-}$, compared with WT (Figure 4). The up‐regulated expression of *OsNAR2.1* and *OsNRT2.3a*,* OsNRT2.2* and *OsNRT2.1* caused the 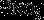 influx rates of *pOsNAR2.1:OsNAR2.1* transgenic lines in 0.2 mm 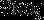, 2.5 mm 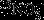 and 1.25 mm NH_4_^15^NO_3_ to increase 32%, 26% and 22%, respectively (Figures 5 and S3).

### Enhanced ${\text{NO}}_{3}^{-}$ uptake promotes ${\text{NH}}_{4}^{+}$ uptake in rice

Kronzucker *et al*. (2000) used ^13^N to show that the presence of ${\text{NO}}_{3}^{-}$ promotes ${\text{NH}}_{4}^{+}$ uptake, accumulation and metabolism in rice. Duan *et al*. (2006) found that increasing ${\text{NO}}_{3}^{-}$ uptake promotes dry weight and ${\text{NO}}_{3}^{-}$ accumulation and assimilation of ${\text{NH}}_{4}^{+}$ and ${\text{NO}}_{3}^{-}$ by ‘Nanguang’, which is an N‐efficient rice cultivar, during the entire growth period. Li *et al*. (2006) showed that supplying ${\text{NH}}_{4}^{+}$ and ${\text{NO}}_{3}^{-}$ enhances *OsAMT1;3*,* OsAMT1;2* and *OsAMT1;1* expression compared with supplying only ${\text{NH}}_{4}^{+}$ or ${\text{NO}}_{3}^{-}$, thereby enhancing ${\text{NH}}_{4}^{+}$ uptake by rice.

High expression of *OsNRT2.3b* in rice improves the pH‐buffering capacity of the rice resulting in less ^15^N‐NH_4_^15^NO_3_ uptake in 5‐min uptake experiment, and more ^15^N‐^15^NH_4_NO_3_ increased uptake at pH 4 and pH 6 (Fan *et al*., 2016).

Our results showed that the influx rates of 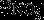 and 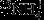 increased 22% and 21%, respectively, in *pOsNAR2.1:OsNAR2.1* transgenic lines in 1.25 mm NH_4_^15^NO_3_ or 1.25 mm ^15^NH_4_NO_3_ (Figure S3), and that the ratio of 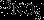 to 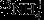 influx into *pOsNAR2.1:OsNAR2.1* transgenic plants was not different from WT in 1.25 mm NH_4_^15^NO_3_ or 1.25 mm ^15^NH_4_NO_3_ (Figure S3). Eventually, the biomass and total nitrogen content of *pOsNAR2.1:OsNAR2.1* transgenic lines increased by 50.7% and 68.9% after 3 weeks in 1.25 mm NH_4_NO_3_ (Figures 2d, 2h and 3h).

### Exogenous of *pOsNAR2.1:OsNAR2.1* transformation in rice enhances ANUE and NRE

During recent years, ${\text{NO}}_{3}^{-}$ transporter gene as a target gene was applied in crop high NUE breeding (Fan *et al*., 2017). For examples, the *OsNRT1.1B* low‐affinity ${\text{NO}}_{3}^{-}$ transporter can increase the *indica* rice NUE by approximately 30% (Hu *et al*., 2015). Fan *et al*. (2016) showed that increased *OsNRT2.3b* expression improved NUE and grain yield by up to 40% in Japonica cultivars. Chen *et al*. (2016) reported the ANUE of *pOsNAR2.1:OsNRT2.1* transgenic plants increased by 28% of in the same background cultivar (Wuyunjing 7) as this experiment. Our present data show that *OsNAR2.1* driven by the native *OsNAR2.1* promoter can produce a relatively higher yield and ANUE in rice plants (Figure 7, Figure S2b and Table S3).

Nitrogen redistribution can be altered by the expression change of some nitrogen use relative gene, such as the autophagy gene ATG8c (Islam *et al*., 2016) and also presents different patterns in different genotypes (Sanchez‐Bragado *et al*., 2017; Souza *et al*., 1998). During rice grain filling, 70‐90% of the nitrogen was re‐distributed from the vegetative organs to the panicles (Yoneyama *et al*., 2016). Dry matter and nitrogen content of *pOsNAR2.1:OsNAR2.1* lines were more than WT plants in the anthesis and maturity stages (Figure 6). Although DMT and NT increased by approximately 21% and 33%, compared to that of WT, DMTE and NTE of *pOsNAR2.1:OsNAR2.1* transgenic plants and WT were not different (Table 3), suggested that dry matter and nitrogen transfer from shoots to grains did not change significantly between *pOsNAR2.1:OsNAR2.1* transgenic plants and WT; thus, the physiological NUE and NHI of *pOsNAR2.1:OsNAR2.1* transgenic plants did not increase (Table 3). NRE and ANUE increased 25% and 22% due to the increase in nitrogen accumulation and grain yield, respectively, at maturity in *pOsNAR2.1:OsNAR2.1* transgenic lines (Table 2; Figure 7a, b).

### Comparison of growth and NUE of *pOsNAR2.1:OsNRT2.1* and *pOsNAR2.1:OsNAR2.1* transgenic lines

Chen *et al*. (2016) reported that co‐expressing *OsNAR2.1* and *OsNRT2.1* in *pOsNAR2.1:OsNRT2.1* transgenic lines increased rice grain yield and ANUE. We also compared field growth between *pOsNAR2.1:OsNRT2.1* and *pOsNAR2.1:OsNAR2.1* transgenic lines. *OsNAR2.1* expressed significantly higher in the culms in the *pOsNAR2.1:OsNAR2.1* lines than in the *pOsNAR2.1:OsNRT2.1* lines, but there was no difference in *OsNRT2.1* expression between them, except at 75 days (Figure 8), which is a key period for grain filling and a critical transition period between rice vegetative and reproductive growth (Zhang *et al*., 2009). *OsNRT2.1* expression and N uptake decreased in the *pOsNAR2.1:OsNAR2.1* lines during the grain filling stage. Postanthesis N uptake decreased in the *pOsNAR2.1:OsNAR2.1* lines compared with the *pOsNAR2.1:OsNRT2.1* plants (Figure 7f). N for rice grain filling comes mainly from accumulation before flowering (Table S4). Although DMTE and NTE did not differ between *pOsNAR2.1:OsNRT2.1* and *pOsNAR2.1:OsNAR2.1* transgenic lines (Table S4), NRE and ANUE of *pOsNAR2.1:OsNAR2.1* transgenic lines were significantly lower than those of *pOsNAR2.1:OsNRT2.1* transgenic lines (Figure 7d, e).

### Designing a genetically modified crop using tissue‐specific expression conferred by selected promoters

Although using either the ubiquitin promoter (*pUbi*) or *OsNAR2.1* promoter (*pOsNAR2.1*) to drive *OsNRT2.1* expression could significantly increase total biomass and grain yield compared with those in WT, ANUE was decreased 17% by *pUbi:OsNRT2.1* expression and increased 28% by *pOsNAR2.1:OsNRT2.1* expression (Chen *et al*., 2016). These opposite effects of different promoters driving *OsNRT2.1* expression on ANUE were caused mainly by altered tissue localization and abundance of *OsNRT2.1* transcripts which may be linked to postflowering transfer of dry matter into grains (Chen *et al*., 2016). Another transformation example of native promoter driving its ORF is *pOsPTR9*:*OsPTR9* transgene in rice with improving on growth, grain yield and NUE (Fang *et al*., 2013). Fang *et al*. (2013) investigated the expression pattern of *OsPTR9* and found that it is regulated by nitrogen sources and light. Although OsPTR9 does not appear to directly transport ${\text{NO}}_{3}^{-}$, its overexpression results in enhanced ${\text{NH}}_{4}^{+}$ uptake, increased grain yield and promoted lateral root formation (Fang *et al*., 2013). These results indicate that expression of genes using specific promoters may be a good approach for plant breeding.

Several phloem ${\text{NO}}_{3}^{-}$ transporters, such as NPF2.13, NPF1.1 and NPF1.2, are responsible for redistributing xylem‐borne ${\text{NO}}_{3}^{-}$ into developing leaves to increase shoot growth (Fan *et al*., 2009; Hsu and Tsay, 2013). Therefore, selecting and applying the promoters of genes specifically expressed in senescing leaves or other source organs could be used to drive phloem‐expressed ${\text{NO}}_{3}^{-}$, transporters, which would decrease residual N in old vegetative organs and increase growth and NUE.

In this experiment, we demonstrated that rice ${\text{NO}}_{3}^{-}$ uptake, yield and NUE of rice were ameliorated by increasing *OsNAR2.1* expression using its native promoter.

## Experimental procedures

### Construction of transgenic rice with *pOsNAR2.1:OsNAR2.1*

The primers in Table S1 amplified the *OsNAR2.1* ORF sequence from the cDNA of *Oryza sativa* L. ssp. Japonica cv. ‘Nipponbare’. The *OsNAR2.1* promoter was amplified from the *pOsNAR2.1‐(1,698 bp):GUS* constructs (Feng *et al*., 2011). The PCR amplification products were ligated into pMD19‐T vector (TaKaRa Bio, Shiga, Japan) independently and after sequencing check we construct the *pOsNAR2.1:OsNAR2.1* plasmid by subcloning. The constructed *pOsNAR2.1:OsNAR2.1* vector is shown in Figure S1 and was transformed into callus of Wuyunjing 7 (*O. sativa* L. ssp. Japonica cv.) by *Agrobacterium tumefaciens* strain EHA105 (Chen *et al*., 2016).

### qRT‐PCR and Southern blot analysis

Total RNA was extracted using TRIzol reagent (Vazyme Biotech Co., Ltd, http://www.vazyme.com). DNase I‐treated total RNAs were subjected to reverse transcription (RT) with HiScript Q RT SuperMix for qPCR (+gDNA wiper) kit (Vazyme Biotech Co.). Triplicate quantitative assays were performed using the AceQ qPCR SYBR Green Master Mix kit (Vazyme Biotech Co.) and a Step One Plus Real‐Time PCR System (Applied Biosystems, Foster City, CA). The relative quantitative calculation of real‐time PCR was described in Chen *et al*. (2016). The primers for PCR are shown in Table S2.

The Southern blot was carried to identify the T‐DNA insertion. The genomic DNA exaction of T2 plant shoots, DNA digestion and hybridization were followed the previous report (Chen *et al*., 2016)

### Western blot

OsNAR2.1 antibody and Western blot process was described in Yan *et al*. (2011). The total protein of 10 g shoots were sampled and 50 μg of each protein was analysed in gel‐loaded buffer and boiled in 10% SDS‐PAGE. Protein transfer to PVDF membrane and incubated with OsActin (1 : 5000), or OsNAR2.1 (1 : 2000) overnight at 4 °C. The membrane was then incubated with the appropriate secondary antibody (1 : 20 000; Pierce), then carries on the chemiluminescence detection (Tang *et al*., 2012; Yan *et al*., 2011).

### Field experiments for harvest yield

The rice plants of T0 to T4 generations, except T3 generation, were cultivated in plots at the Experimental Site of Nanjing Agricultural University, Nanjing, with subtropical climate from May to October in a year. For T3 generation, transgenic lines were tested in plots of Experiment Site of Sanya Nanjing Agricultural University with tropical climate from December to April. Soil properties in Nanjing field experiment were described as before (Chen *et al*., 2016).

T2–T4 generation *pOsNAR2.1:OsNAR2.1* and wild‐type plants were planted in three plots with 300 kg N/ha and without nitrogen fertilizer as blank control. The plots were 2 × 2 m in size, and the seedlings were planted in a 10 × 10 array. During rice flowering and mature stages, we collected samples from each plot for further analysis. Random four replicates (each replicate with four individual plants) from each plot were selected within the plots free from the edges, and therefore, the data of total 16 individual plants were pulled into mean value of each plot (Chen *et al*., 2016).

The agronomic characters of T4 generation plant height, total tiller number per plant, grain weight per panicle, grain number per panicle, seed setting rate, panicle length, 1000‐grain weight, yield and biomass per plant were measured at the maturity stage under 300 kg N/ha N fertilizer condition.

### Dry weight, total nitrogen measurement and calculation of nitrogen use efficiency

We harvested T4 generation shoot samples from the field to analyse biomass and nitrogen under 300 kg N/ha fertilizer condition according to our previous method (Chen *et al*., 2016) DMTE and NTE were calculated according to Chen *et al*. (2016). DMT (kg/ha) = DMA–(DMM–GY); CPAY (%) = (DMT/GY) × 100%; DMTE (%) = (DMT/DMA) × 100%; HI (%) = (GY/DMM) × 100%; The NUE method was used for the calculation as described by Chen *et al*. (2016). ANUE (kg/kg) = (GY–GY of zero‐N plot)/N supply; PNUE (kg/kg) = (GY–GY of zero‐N plot)/TNAM; NRE (%) = (TNAM–TNAM of zero‐N plot)/N supply; PANU (kg/ha) = TNAM–TNAA; NHI (%) = (GNAM/TNAM) × 100%; NT (kg/ha) = TNAA–(TNAM–GNAM); CPNGN (%) = (NT/GNAM)  × 100%; NTE (%) = (NT/TNAA) × 100%.

### Determination of total N content, root ^15^N‐${\text{NO}}_{3}^{-}$ influx rate and ^15^N‐${\text{NH}}_{4}^{+}$ influx rate in WT and transgenic seedlings

WT and transgenic rice seedlings were grown in the solution containing 1 mm ${\text{NH}}_{4}^{+}$ in IRRI solution for 2 weeks and then transferred in different forms of nitrogen for 3 additional weeks. The nitrogen treatments in this experiment included in 2.5 mm ${\text{NH}}_{4}^{+}$, 0.2 mm ${\text{NO}}_{3}^{-}$, 2.5 mm ${\text{NO}}_{3}^{-}$ and 1.25 mm NH_4_NO_3_. The biomass and nitrogen concentration were measured for each line (*n* = 4 plants) under each N treatment after 3‐week treatment.

For root ^15^N uptake experiment, new rice seedlings were grown in 1 mm ${\text{NH}}_{4}^{+}$ for 3 weeks and then were nitrogen starved for 1 week before ^15^N uptake. 2.5 mm 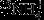, 0.2 mm 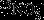, 2.5 mm 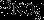, 1.25 mm ^15^NH_4_NO_3_, 1.25 mm NH_4_^15^NO_3_ or 1.25 mm ^15^NH_4_^15^NO_3_ (atom % ^15^N: 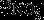, 99%; 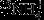, 99%) was used, and the ^15^N influx rate was calculated following the method in Tang *et al*. (2012).

### Statistical analysis

The single‐factor analysis of variance (ANOVA) and Tukey's test data analysis were applied in our data statistical analysis (Chen *et al*. (2016).

## Supporting information


**Figure S1** Diagram of *pOsNAR2.1:OsNAR2.1* constructs. RB, right border; LB, left border; *pOsNAR2.1*,* OsNAR2.1* promoter; 35S, cauliflower mosaic virus 35S promoter; NOS, nopaline synthase terminator.
**Figure S2** Characterization of T1 generation *pOsNAR2.1:OsNAR2.1* transgenic lines. (a) qRT‐PCR analysis of endogenous the expression of *OsNAR2.1* in culms of wild type and *pOsNAR2.1:OsNAR2.1* transgenic lines. Error bars: SE (*n* = 3 plants). (b) Yield and biomass per plant from wild‐type and *pOsNAR2.1:OsNAR2.1* transgenic lines grown in the field. Error bars: SE (*n* = 5 plants).
**Figure S3** Ratio of 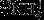 to 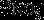 influx in wild‐type and *pOsNAR2.1:OsNAR2.1* transgenic lines in 1.25 mm NH_4_NO_3_. WT and transgenic seedlings were grown in 1 mm ${\text{NH}}_{4}^{+}$ for 3 weeks and nitrogen starved for 1 week. 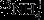 or 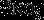 influx was measured at (a) 1.25 mm ^15^NH_4_NO_3_ or (b) 1.25 mm NH_4_^15^NO_3_ for 5 min. DW, dry weight. (c) The 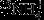 to 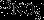 influx ratios with 1.25 mm NH_4_NO_3_ in the roots of wild‐type and *pOsNAR2.1:OsNAR2.1* lines (Ox1, Ox2, and Ox3) are presented. Error bars: SE (*n* = 4 plants). The different letters indicate a significant difference between the transgenic line and the WT (*P* < 0.05, one‐way ANOVA).
**Figure S4** Expression ratios of *OsNRT2.1* to *OsNAR2.1* in culms of transgenic lines and wild type. The *pOsNAR2.1:OsNRT2.1* lines (O6, O7 and O8), *pOsNAR2.1:OsNAR2.1* lines (Ox1, Ox2, and Ox3) and wild type are presented.
**Table S1** Primers for amplification *OsNAR2.1* ORF.
**Table S2** Primers used for qRT‐PCR.

**Table S3** Comparison of dry weight, grain yield, and ANUE between the wild‐type and *pOsNAR2.1:OsNAR2.1* transgenic lines in the T2–T4 generations. *n* = 3 plots for each mean. The different letters indicate a significant difference between the transgenic line and the WT (*P* < 0.05, one‐way ANOVA).
**Table S4** Increased nitrogen‐use efficiency in *pOsNAR2.1:OsNAR2.1* and *pOsNAR2.1:OsNRT2.1* transgenic lines relative to wild type. Statistical analysis of data from T4 generation; *n* = 3 for each mean. The different letters indicate a significant difference between the transgenic line and the WT (*P* < 0.05, one‐way ANOVA).
