# Supplementary material for: pOsNAR2.1:OsNAR2.1 expression enhances nitrogen uptake efficiency and grain yield in transgenic rice plants
Source: Plant Biotechnol J. 2017 Mar 29;15(10):1273–83. doi: 10.1111/pbi.12714 (PMC5595721; doi:10.1111/pbi.12714)
Supplement: Supplementary file 1 — Figure S1 Diagram of pOsNAR2.1:OsNAR2.1 constructs. RB, right border; LB, left border; pOsNAR2.1, OsNAR2.1 promoter; 35S, cauliflower mosaic virus 35S promoter; NOS, nopaline synthase terminator. Figure S2 Characterization of T1 generation pOsNAR2.1:OsNAR2.1 transgenic lines. (a) qRT‐PCR analysis of endogenous the expression of OsNAR2.1 in culms of wild type and pOsNAR2.1:OsNAR2.1 transgenic lines. Error bars: SE (n = 3 plants). (b) Yield and biomass per plant from wild‐type and pOsNAR2.1:OsNAR2.1 transgenic lines grown in the field. Error bars: SE (n = 5 plants). Figure S3 Ratio of to influx in wild‐type and pOsNAR2.1:OsNAR2.1 transgenic lines in 1.25 mm NH4NO3. WT and transgenic seedlings were grown in 1 mm NH4+ for 3 weeks and nitrogen starved for 1 week. or influx was measured at (a) 1.25 mm 15NH4NO3 or (b) 1.25 mm NH4 15NO3 for 5 min. DW, dry weight. (c) The to influx ratios with 1.25 mm NH4NO3 in the roots of wild‐type and pOsNAR2.1:OsNAR2.1 lines (Ox1, Ox2, and Ox3) are presented. Error bars: SE (n = 4 plants). The different letters indicate a significant difference between the transgenic line and the WT (P < 0.05, one‐way ANOVA). Figure S4 Expression ratios of OsNRT2.1 to OsNAR2.1 in culms of transgenic lines and wild type. The pOsNAR2.1:OsNRT2.1 lines (O6, O7 and O8), pOsNAR2.1:OsNAR2.1 lines (Ox1, Ox2, and Ox3) and wild type are presented. Table S1 Primers for amplification OsNAR2.1 ORF. Table S2 Primers used for qRT‐PCR. Table S3 Comparison of dry weight, grain yield, and ANUE between the wild‐type and pOsNAR2.1:OsNAR2.1 transgenic lines in the T2–T4 generations. n = 3 plots for each mean. The different letters indicate a significant difference between the transgenic line and the WT (P < 0.05, one‐way ANOVA). Table S4 Increased nitrogen‐use efficiency in pOsNAR2.1:OsNAR2.1 and pOsNAR2.1:OsNRT2.1 transgenic lines relative to wild type. Statistical analysis of data from T4 generation; n = 3 for each mean. The different letters indicate a significant differe [file PBI-15-1273-s001.ppt]

## Slide 1
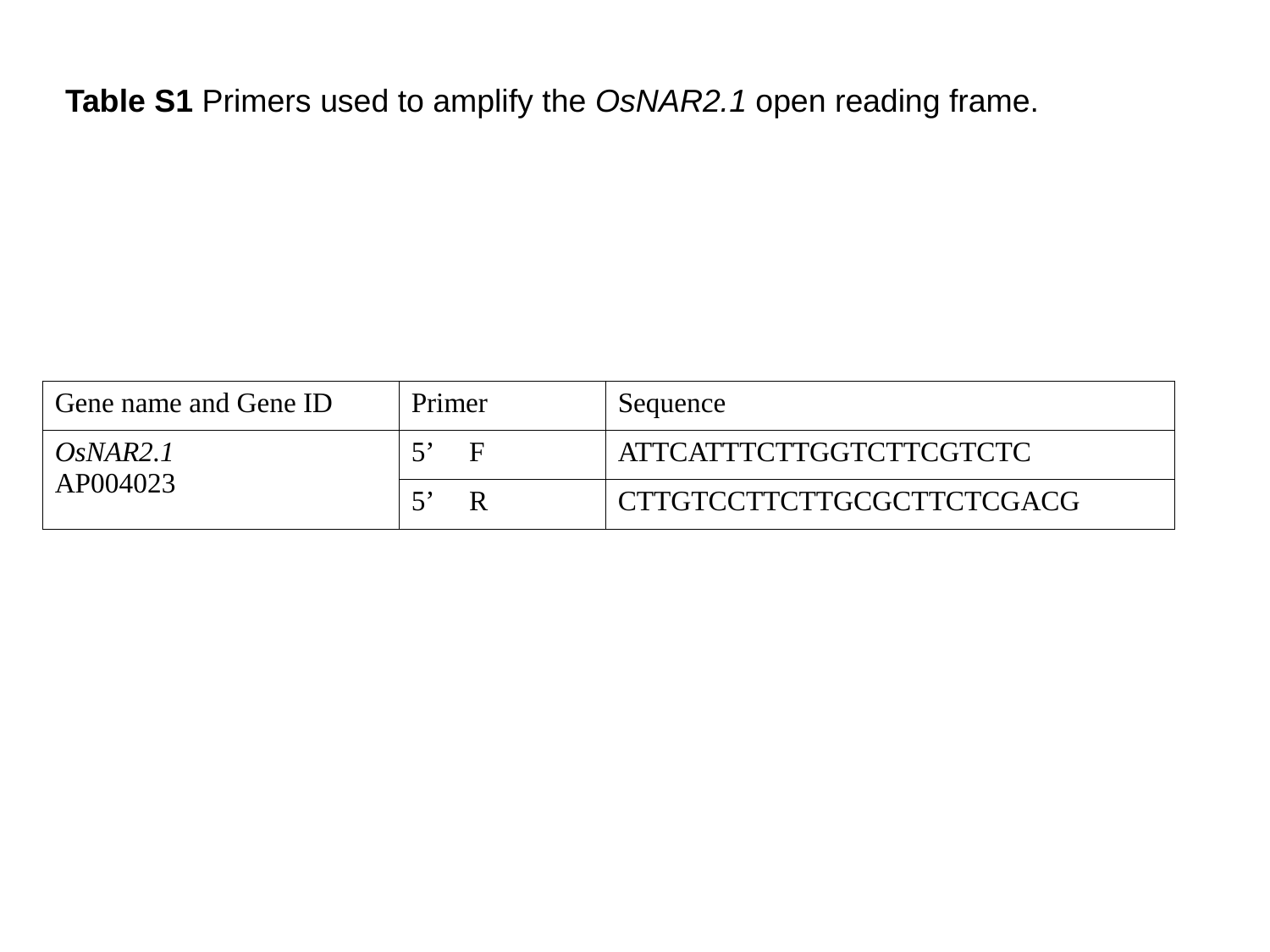

Table S1 Primers used to amplify the OsNAR2.1 open reading frame.
| Gene name and Gene ID | Primer | Sequence |
| --- | --- | --- |
| OsNAR2.1 AP004023 | 5’（F） | ATTCATTTCTTGGTCTTCGTCTC |
| | 5’（R） | CTTGTCCTTCTTGCGCTTCTCGACG |

## Slide 2
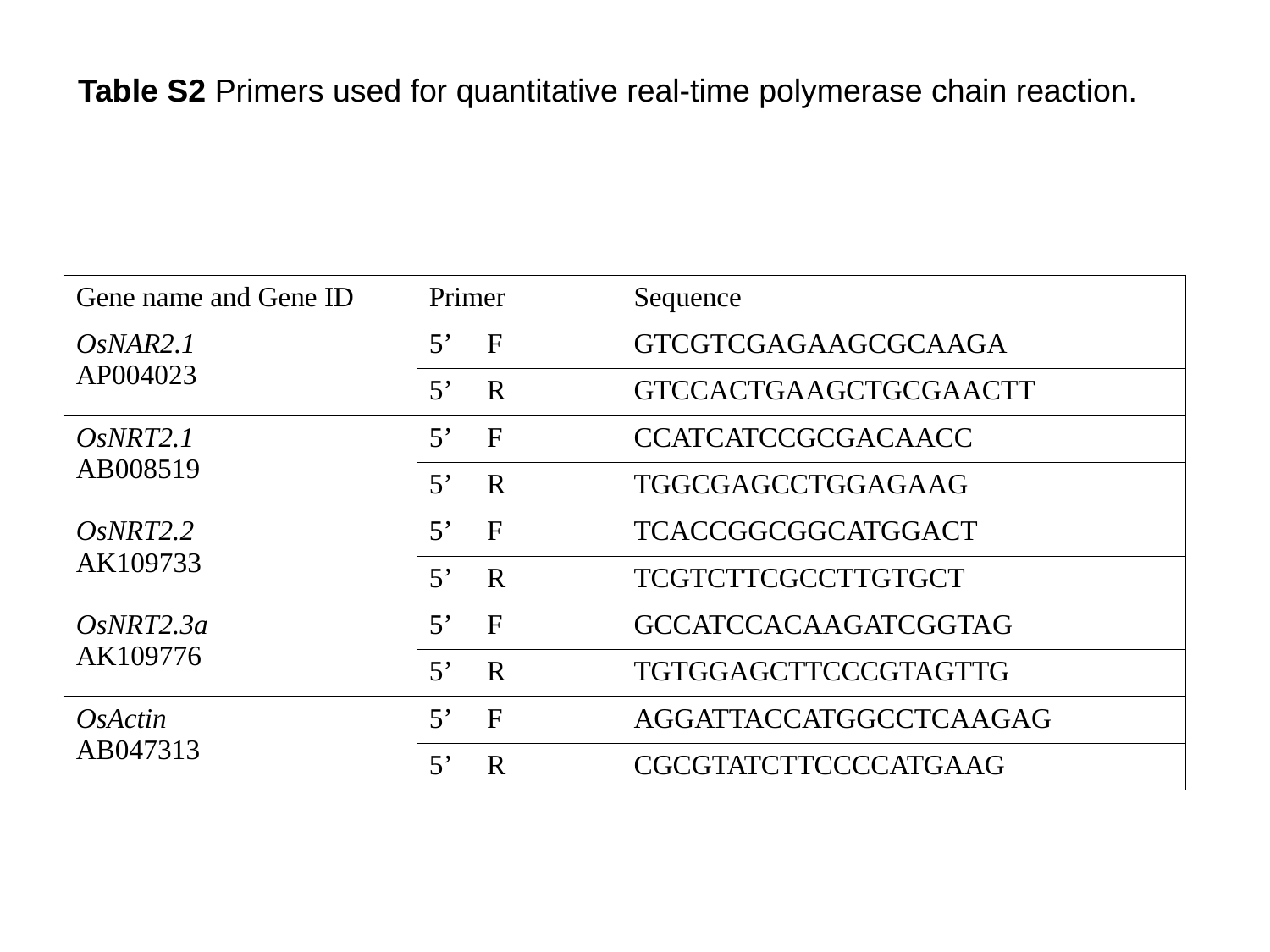

Table S2 Primers used for quantitative real-time polymerase chain reaction.
| Gene name and Gene ID | Primer | Sequence |
| --- | --- | --- |
| OsNAR2.1 AP004023 | 5’（F） | GTCGTCGAGAAGCGCAAGA |
| | 5’（R） | GTCCACTGAAGCTGCGAACTT |
| OsNRT2.1 AB008519 | 5’（F） | CCATCATCCGCGACAACC |
| | 5’（R） | TGGCGAGCCTGGAGAAG |
| OsNRT2.2 AK109733 | 5’（F） | TCACCGGCGGCATGGACT |
| | 5’（R） | TCGTCTTCGCCTTGTGCT |
| OsNRT2.3a AK109776 | 5’（F） | GCCATCCACAAGATCGGTAG |
| | 5’（R） | TGTGGAGCTTCCCGTAGTTG |
| OsActin AB047313 | 5’（F） | AGGATTACCATGGCCTCAAGAG |
| | 5’（R） | CGCGTATCTTCCCCATGAAG |

## Slide 3
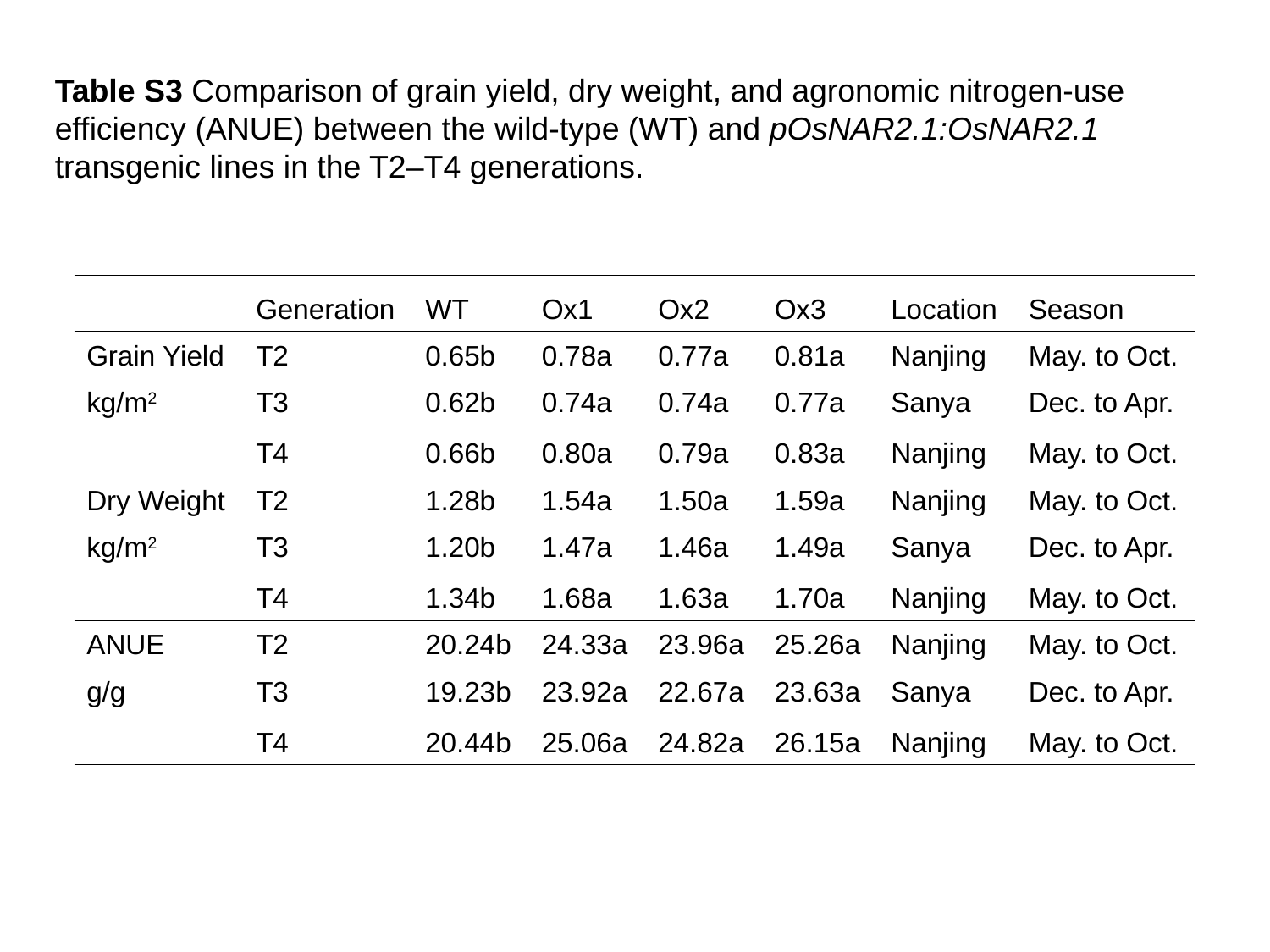

Table S3 Comparison of grain yield, dry weight, and agronomic nitrogen-use efficiency (ANUE) between the wild-type (WT) and pOsNAR2.1:OsNAR2.1 transgenic lines in the T2–T4 generations.
| | Generation | WT | Ox1 | Ox2 | Ox3 | Location | Season |
| --- | --- | --- | --- | --- | --- | --- | --- |
| Grain Yield | T2 | 0.65b | 0.78a | 0.77a | 0.81a | Nanjing | May. to Oct. |
| kg/m2 | T3 | 0.62b | 0.74a | 0.74a | 0.77a | Sanya | Dec. to Apr. |
| | T4 | 0.66b | 0.80a | 0.79a | 0.83a | Nanjing | May. to Oct. |
| Dry Weight | T2 | 1.28b | 1.54a | 1.50a | 1.59a | Nanjing | May. to Oct. |
| kg/m2 | T3 | 1.20b | 1.47a | 1.46a | 1.49a | Sanya | Dec. to Apr. |
| | T4 | 1.34b | 1.68a | 1.63a | 1.70a | Nanjing | May. to Oct. |
| ANUE | T2 | 20.24b | 24.33a | 23.96a | 25.26a | Nanjing | May. to Oct. |
| g/g | T3 | 19.23b | 23.92a | 22.67a | 23.63a | Sanya | Dec. to Apr. |
| | T4 | 20.44b | 25.06a | 24.82a | 26.15a | Nanjing | May. to Oct. |

## Slide 4
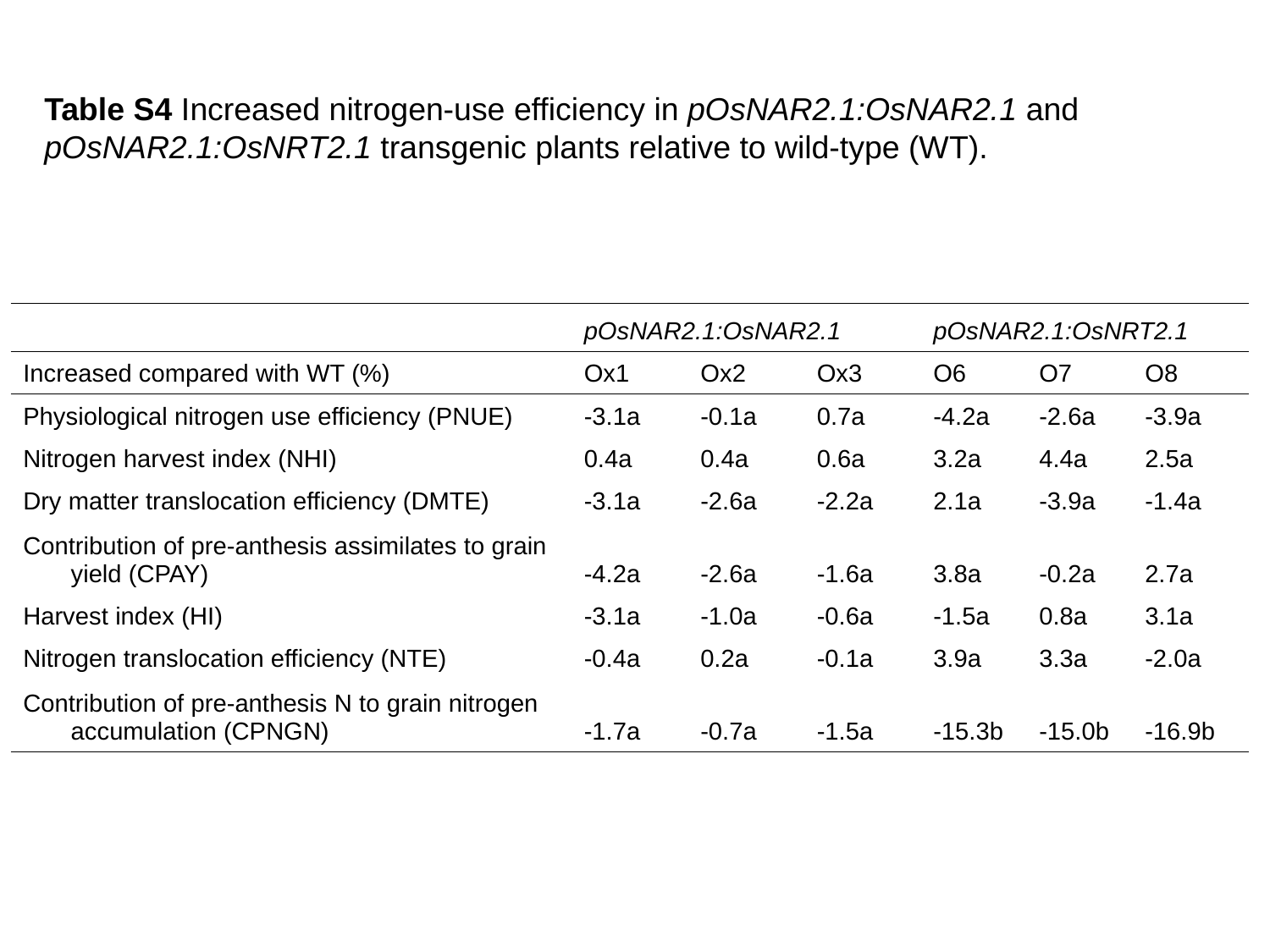

Table S4 Increased nitrogen-use efficiency in pOsNAR2.1:OsNAR2.1 and pOsNAR2.1:OsNRT2.1 transgenic plants relative to wild-type (WT).
| | pOsNAR2.1:OsNAR2.1 | | | pOsNAR2.1:OsNRT2.1 | | |
| --- | --- | --- | --- | --- | --- | --- |
| Increased compared with WT (%) | Ox1 | Ox2 | Ox3 | O6 | O7 | O8 |
| Physiological nitrogen use efficiency (PNUE) | -3.1a | -0.1a | 0.7a | -4.2a | -2.6a | -3.9a |
| Nitrogen harvest index (NHI) | 0.4a | 0.4a | 0.6a | 3.2a | 4.4a | 2.5a |
| Dry matter translocation efficiency (DMTE) | -3.1a | -2.6a | -2.2a | 2.1a | -3.9a | -1.4a |
| Contribution of pre-anthesis assimilates to grain yield (CPAY) | -4.2a | -2.6a | -1.6a | 3.8a | -0.2a | 2.7a |
| Harvest index (HI) | -3.1a | -1.0a | -0.6a | -1.5a | 0.8a | 3.1a |
| Nitrogen translocation efficiency (NTE) | -0.4a | 0.2a | -0.1a | 3.9a | 3.3a | -2.0a |
| Contribution of pre-anthesis N to grain nitrogen accumulation (CPNGN) | -1.7a | -0.7a | -1.5a | -15.3b | -15.0b | -16.9b |

## Slide 5
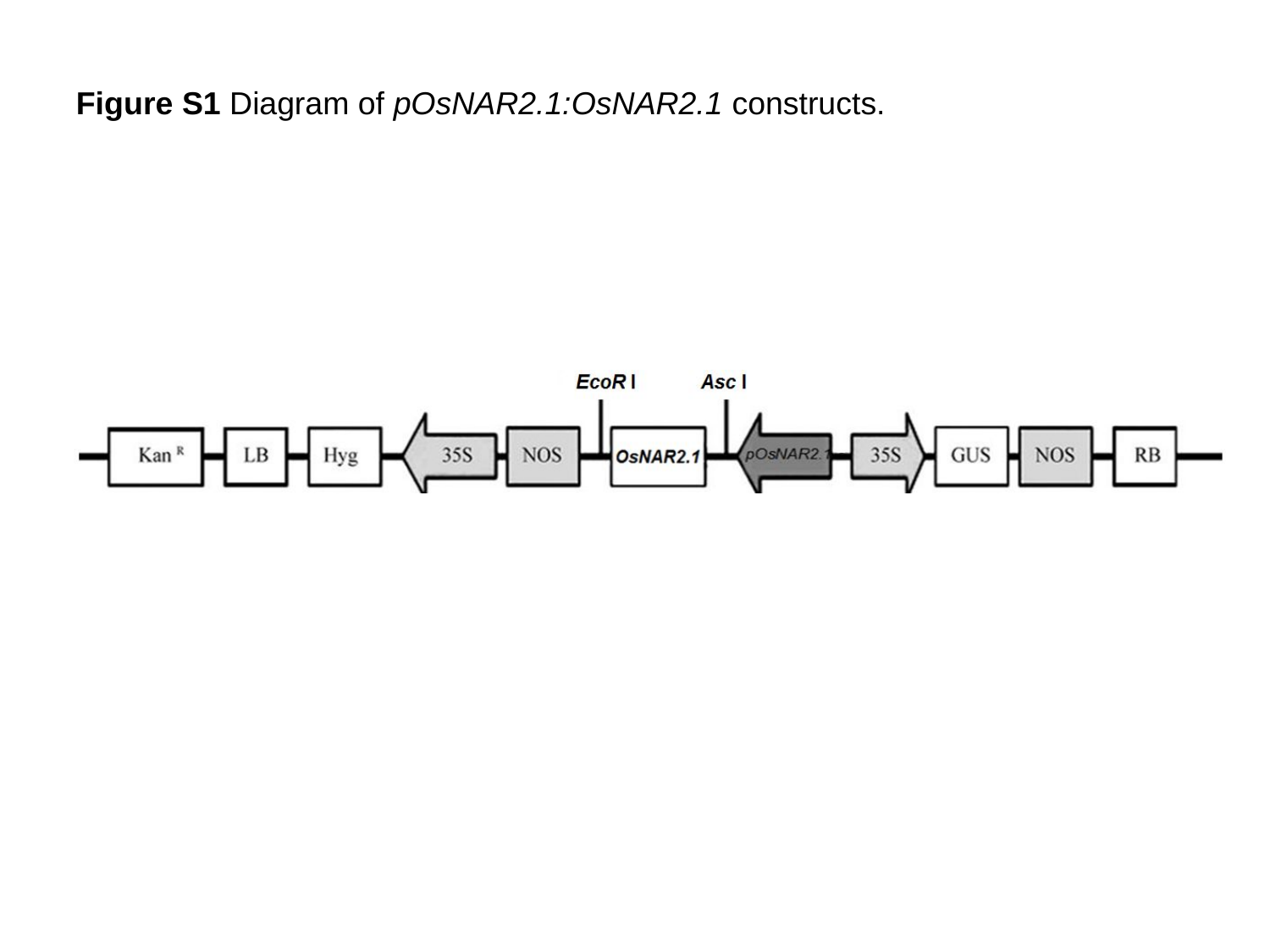

Figure S1 Diagram of pOsNAR2.1:OsNAR2.1 constructs.

## Slide 6
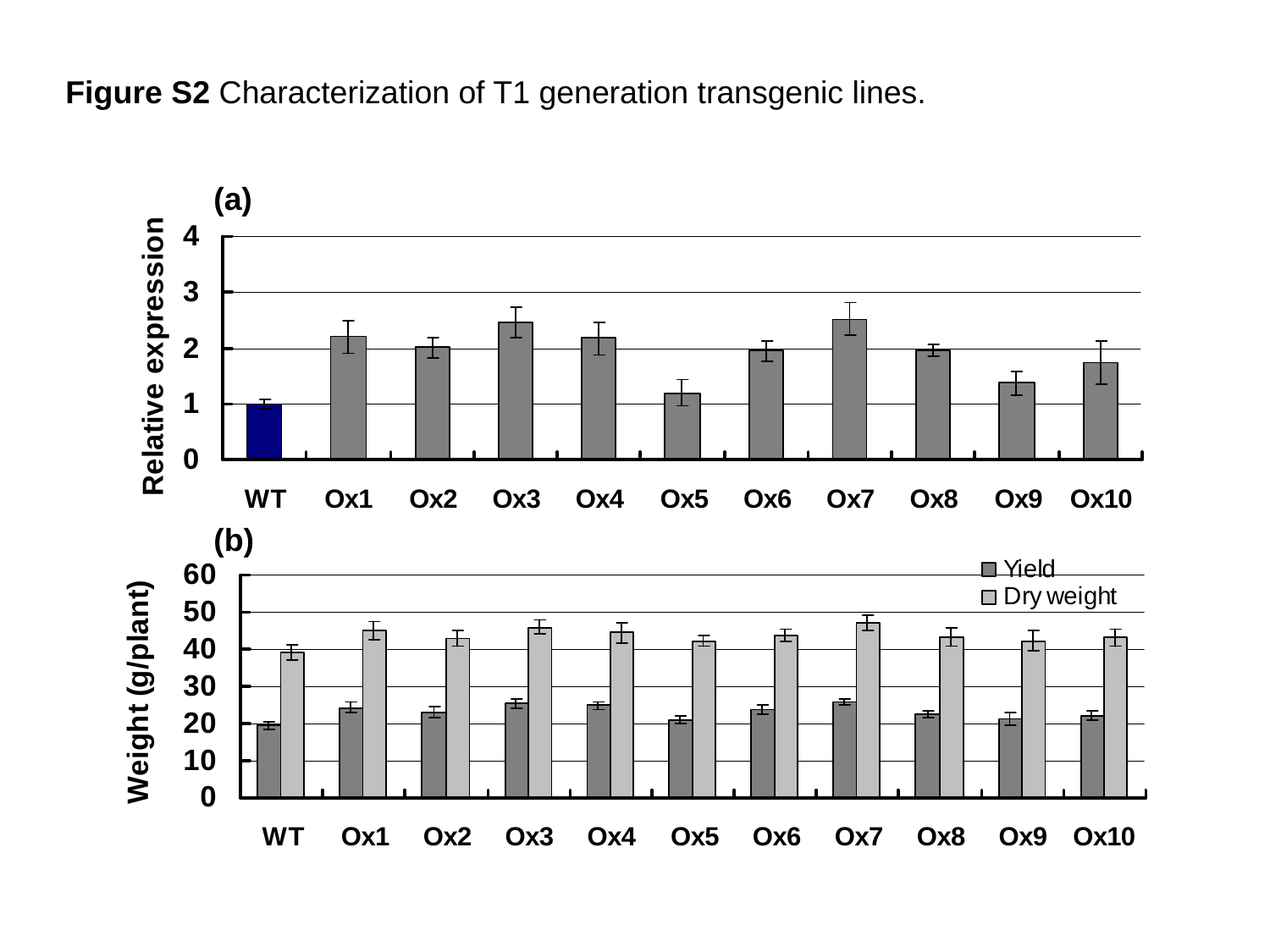

Figure S2 Characterization of T1 generation transgenic lines.
(a)
(b)

## Slide 7
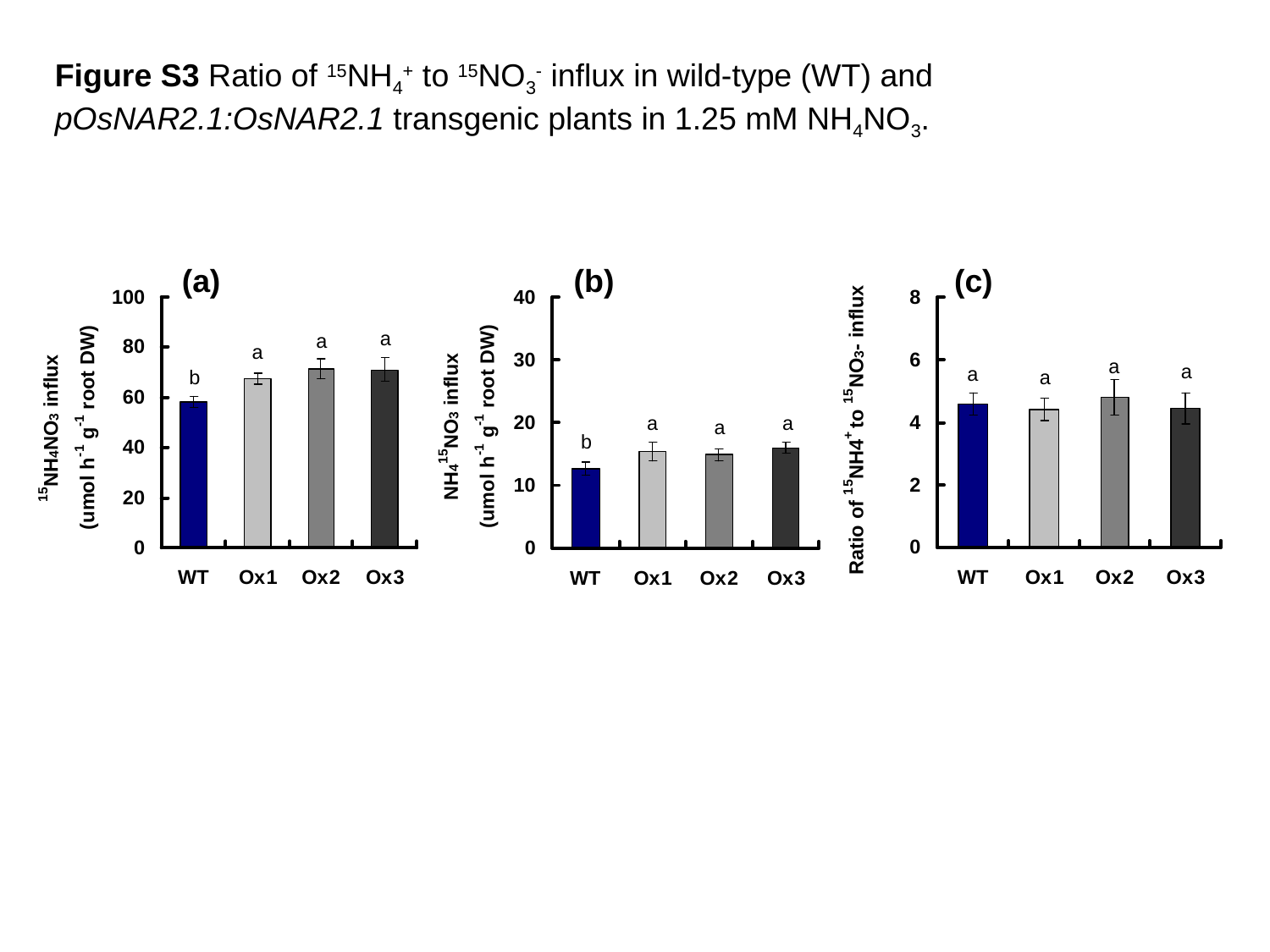

Figure S3 Ratio of 15NH4+ to 15NO3- influx in wild-type (WT) and pOsNAR2.1:OsNAR2.1 transgenic plants in 1.25 mM NH4NO3.
(a)
(b)
(c)

## Slide 8
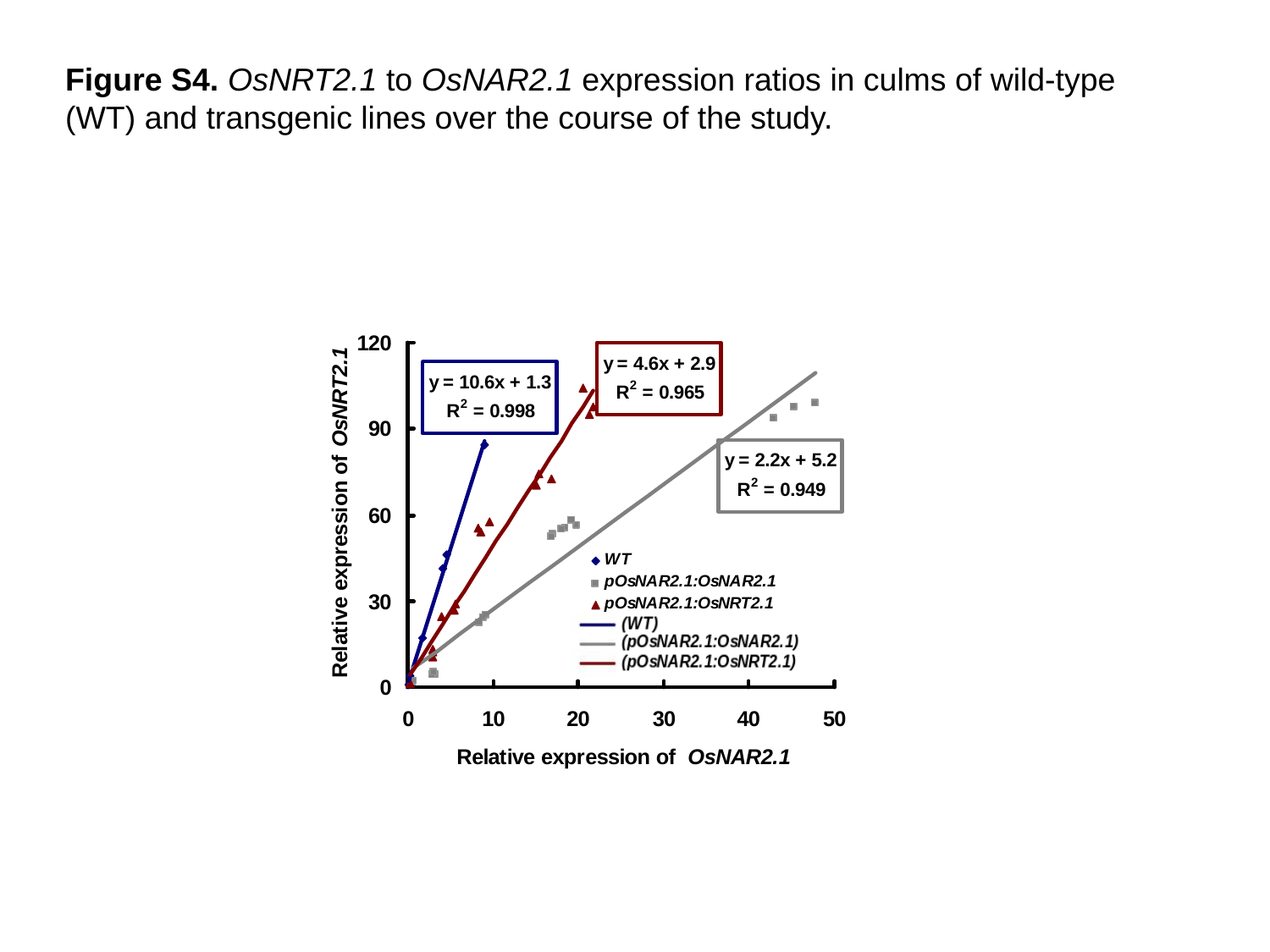

Figure S4. OsNRT2.1 to OsNAR2.1 expression ratios in culms of wild-type (WT) and transgenic lines over the course of the study.
